# Differentiated metabolomic profiling reveals plasma amino acid signatures for primary glomerular disease

**DOI:** 10.1007/s00726-024-03407-4

**Published:** 2024-07-18

**Authors:** Jiao Wang, Chunyu Zhou, Liqian Lu, Shoujun Wang, Qing Zhang, Zhangsuo Liu

**Affiliations:** 1 Department of geriatric endocrinology, the First Affiliated Hospital of Zhengzhou University, Zhengzhou, 450052 P. R. China; 2 Blood Purification Center, the First Affiliated Hospital of Zhengzhou University, Zhengzhou, 450052 P. R. China; 3 Department of endocrinology, the First Affiliated Hospital of Zhengzhou University, Zhengzhou, 450052 P. R. China; 4 Traditional Chinese Medicine Integrated Department of Nephrology, The First Affiliated Hospital of Zhengzhou University, Zhengzhou, 450052 P. R. China; 5 Research Institute of Nephrology, Zhengzhou University, Zhengzhou, 450052 P. R. China; 6 Henan Province Research Center For Kidney Disease, Zhengzhou, 450052 P. R. China

**Keywords:** Primary glomerular disease_1_, Amino acids_2_, Metabolomics_3_, LC-MS/MS_4_, Diagnostic model_5_

## Abstract

Primary glomerular disease (PGD) is an idiopathic cause of renal glomerular lesions that is characterized by proteinuria or hematuria and is the leading cause of chronic kidney disease (CKD). The identification of circulating biomarkers for the diagnosis of PGD requires a thorough understanding of the metabolic defects involved. In this study, ultra-high performance liquid chromatography–tandem mass spectrometry was performed to characterize the amino acid (AA) profiles of patients with pathologically diagnosed PGD, including minimal change disease (MCD), focal segmental glomerular sclerosis (FSGS), membranous nephropathy, and immunoglobulin A nephropathy. The plasma concentrations of asparagine and ornithine were low, and that of aspartic acid was high, in patients with all the pathologic types of PGD, compared to healthy controls. Two distinct diagnostic models were generated using the differential plasma AA profiles using logistic regression and receiver operating characteristic analyses, with areas under the curves of 1.000 and accuracies up to 100.0% in patients with MCD and FSGS. In conclusion, the progression of PGD is associated with alterations in AA profiles, The present findings provide a theoretical basis for the use of AAs as a non-invasive, real-time, rapid, and simple biomarker for the diagnosis of various pathologic types of PGD.

**Supplementary Information:**

The online version contains supplementary material available at 10.1007/s00726-024-03407-4.

## Introduction

The global median prevalence of chronic kidney disease (CKD) is now 9.5%(Bello et al. 2024). Primary glomerular disease (PGD) is one of the major causes of CKD (Webster et al. 2017), accounting for approximately 40% of cases of end-stage renal disease (ESRD) worldwide. PGD refers to the renal disease consisting predominantly of glomerular lesions, characterized by proteinuria or hematuria, the cause of which is unknown. The clinical classifications of PGD are not independent or specific; instead PGD represents a syndrome of multiple types of glomerular injury. Notably, pathological diagnosis is essential for clinical diagnostic and prognostic of PGD because transformation could occur among different clinical classifications as the disease progression. Renal biopsy remains the gold-standard method of achieving a definitive diagnosis, assigning a prognosis, and determining the most appropriate treatment of PGD, but the procedure is associated with a low, but not negligible, risk of mortality (Bramham et al. 2009), as well as high risks of hematuria and perirenal hematoma (Manno et al. 2004). Therefore, there is an urgent need for the identification of non-invasive biomarkers to aid the diagnosis and systemic therapy of PGD.

The high-throughput, high phenotyping enabled by metabolomics in kidney disease illustrated the rapid translation of metabolite data for predictive and diagnostic aims (Aronov et al. 2011; Kalim and Rhee 2017; Tang et al. 2015). Amino acid (AA) metabolism is dysregulated in kidney disease, and has been a key subject of recent research (Wang et al. 2019). Abe et al. has reported that phenyl sulfate, a tyrosine metabolite derived from gut microbes, may be involved in the etiology of diabetic kidney disease (DKD)(Kikuchi et al. 2019). In addition, the metabolic homeostasis of histidine, tryptophan, methionine, glutamine, tyrosine, and branched-chain AAs has been shown to be closely related to the progression of kidney dysfunction in DKD (Liu et al. 2022). The accumulation of AAs, the fundamental components of proteins (Descamps et al. 2019; Nagata et al. 2023), increases kidney volume and causes both glomerular enlargement and tubular hypertrophy (Friedman 2004). Previously, we have also shown that abnormal AA profiles in the plasma, urine, and saliva of patients with DKD are involved in the decline in kidney function (Wang et al. 2023; Zhou et al. 2021). However, there have been few studies of the AA profiles of patients with PGD or investigations of the relationships between AAs and the specific pathologic types of PGD.

In the present study, we aimed to characterize the plasma profiles of the 20 AAs in patients with biopsy-diagnosed PGD, including minimal change disease (MCD), focal segmental glomerular sclerosis (FSGS), membranous nephropathy (MN), and immunoglobulin A nephropathy (IgAN), using a method based on ultra-high performance liquid chromatography–tandem spectrometry (UPLC-MS/MS). We then established distinct diagnostic models based on the plasma AA profiles of patients with different pathologic classifications. These diagnostic models may provide an innovative means of diagnosing specific pathologic forms of PGD in patients with PGD.

## Materials and methods

### Study design and participants

This study was approved by the Ethics Committee of the First Affiliated Hospital of Zhengzhou University (2021-KY-0477-003) and conformed to the tenets of the Declaration of Helsinki 1964 and its later amendments. Written informed consent was obtained from all the participants. Patients with newly diagnosed PGD and healthy controls were recruited from the Department of Nephrology and the Department of Physical Examination, respectively, at the First Affiliated Hospital of Zhengzhou University. General characteristics of patients and healthy individuals were collected from the electronic medical records of our hospital. Table 1 lists the general characteristics of the participants in each group.

**Table 1:** General characteristics of the study population

	CON (*n* = 30)	MCD (*n* = 30)	FSGS (*n* = 30)	MN (*n* = 30)	IgAN (*n* = 30)	P1	P2	P3	P4
Age, year	49.6 ± 8.4	33.9 ± 15.8	38.4 ± 15.2	46.9 ± 11.2	39.3 ± 11.4	< 0.001	0.001	0.430	0.002
Male/Female, n	16/14	18/12	18/12	20/10	17/13	0.605	0.605	0.301	0.796
Hb, g/L	145.8 ± 12.3	128.6 ± 16.2	123.4 ± 19.5	133.9 ± 13.4	119.8 ± 23.1	< 0.001	< 0.001	0.009	< 0.001
Alb, g/L	49.8 ± 3.2	21.9 ± 4.6	33.6 ± 10.9	29.7 ± 7.8	41.2 ± 5.4	< 0.001	< 0.001	< 0.001	< 0.001
Scr, µM	63.5 ± 8.8	119.5 ± 105.4	119.4 ± 56.9	74.1 ± 15.5	134.4 ± 96.4	0.002	0.002	0.552	< 0.001
Bun, mM	5.3 ± 1.1	6.8 ± 4.3	8.7 ± 3.4	5.7 ± 1.2	8.1 ± 4.3	0.056	< 0.001	0.613	0.001
TC, mM	4.3 ± 0.6	8.5 ± 2.5	5.9 ± 2.3	6.6 ± 1.9	4.3 ± 1.1	< 0.001	0.001	< 0.001	0.882
TG, mM	1.1 ± 0.5	2.4 ± 1.1	2.5 ± 1.1	2.5 ± 1.9	1.7 ± 0.8	< 0.001	< 0.001	< 0.001	0.066
LDL-C, mM	2.3 ± 0.8	6.8 ± 4.9	3.5 ± 2.0	4.5 ± 1.7	2.7 ± 1.0	< 0.001	0.004	< 0.001	0.265
HDL-C, mM	1.3 ± 0.4	1.6 ± 0.6	1.2 ± 0.3	1.2 ± 0.3	1.1 ± 0.3	0.009	0.101	0.145	0.030
UAER, g/24 h	NA	8.1 ± 3.2	4.6 ± 3.3	6.0 ± 4.5	2.0 ± 1.5	< 0.001	< 0.001	< 0.001	< 0.001
eGFR, mL/min/1.73m^2^	112.5 ± 15.7	87.5 ± 36.7	78.4 ± 37.1	101.5 ± 13.9	72.9 ± 36.3	0.002	< 0.001	0.159	< 0.001

UAER values were not tested for healthy individuals in regular health examination

Continuous variables are presented as mean ± SD and were compared with the one-way ANOVA test. Categorical variables were compared by Chi-square test or Fisher’s exact test and presented as counts. P1: MCD vs. CON group; P2: FSGS vs. CON group; P3: MN vs. CON group; P4: IgAN vs. CON group; Alb, albumin; Bun, blood urea nitrogen; CON, healthy controls; eGFR, estimated glomerular filtration rate; FSGS, focal segmental glomerular sclerosis; Hb, hemoglobin; HDL-C, high density lipoprotein cholesterol; IgAN, IgA nephropathy; LDL-C, low density lipoprotein cholesterol; MCD, minimal change disease; MN membranous nephropathy; NA, not available; Scr, serum creatinine; TC, total cholesterol; TG, triglyceride; UAER, urinary albumin excretion rate

The calculation formula of eGFR is the Cockcroft-Gault formula (C-G formula), which is calculated as follows: Ccr= (140- age)×body weight (kg) /72×Scr (mg/dl) or Ccr=[(140- age)×body weight (kg)]/[0.818×Scr (umol/L)]. (Ccr: endogenous creatinine clearance; Scr: Serum creatinine, serum creatinine unit conversion: 1 mg/dl = 88.4umol/L)

The inclusion criteria for the patients with PGD were as follows: (1) age 18–75 years old; (2) a pathologic diagnosis made by the Renal Pathology Laboratory of the First Affiliated Hospital of Zhengzhou University; and (3) the absence of any other clinically diagnosed serious disease, including, but not limited to, cancer, neurological, digestive, psychiatric, and infectious diseases. The inclusion criteria for the healthy controls were as follows: (1) age 18–75 years; (2) lack of a self-reported or clinical diagnosis of any severe disease, including, but not limited to cancer, neurological, renal, digestive, psychiatric, or infectious disease within the preceding 3 months; and (3) the absence of within the 3 months preceding blood sample collection.

### Preparation of samples and standards for metabolomic profiling

Two-mL blood samples were obtained from volunteers after overnight fasting under aseptic conditions into anticoagulant tubes containing EDTA, centrifuged at 1,500 rpm for 10 min at 4 °C, and the plasma samples obtained were stored at − 80 °C until UPLC-MS/MS analysis.

Stock solutions of AAs were prepared by dissolving the compounds in water or dimethyl sulfoxide at concentrations ranging from 1 to 100 mM and stored in brown volumetric flasks at − 80℃ until use. An isotope-labeled AA mix in acetonitrile (ACN) was prepared at 100 nM for use as an internal standard (IS). In addition, working solutions of the AAs were prepared by diluting stock solutions to 10–1,000 nM concentrations using ACN. The plasma samples and standards were then subjected to a protein precipitation extraction method.

### Metabolomic profiling of AAs in samples

The concentrations of AAs were measured in samples using UPLC-MS/MS in positive-ion, multiple reaction monitoring mode on a UPLC-30ADvp series UPLC instrument (Shimadzu, Kyoto, Japan) with an SIL-30-AC autosampler, a CTO-20AC column oven, and an API 6500 triple-quadrupole source (Applied Biosystems Sciex, Toronto, ON, Canada). Supplemental Tables 1 and 2 list the detailed UPLC-MS/MS conditions used for AAs and isotope-labeled AAs, and Supplemental Table 3 shows the gradient program used for liquid chromatography. Analyst v1.6.2 software was used for data acquisition and processing.

### Statistical analysis

The data obtained during the study were inserted into a spreadsheet, then frequencies, percentages, means, standard deviations (SDs), medians, and minimum and maximum values were calculated. Student’s *t*-test and one-way ANOVA were used to compare datasets between groups. In addition, the areas under the receiver operating characteristic (ROC) curves (AUCs), cut-off values, sensitivities, specificities, and accuracies were calculated, to evaluate the predictive performances of key AAs for PGD. *P* < 0.05 was regarded as indicating statistical significance. Data analyses were performed using SPSS v.21.0 (IBM, Inc., Armonk, NY, USA).

## Results

### Plasma profiles of 20 AAs in the participants with PGD

**Fig. 1:**
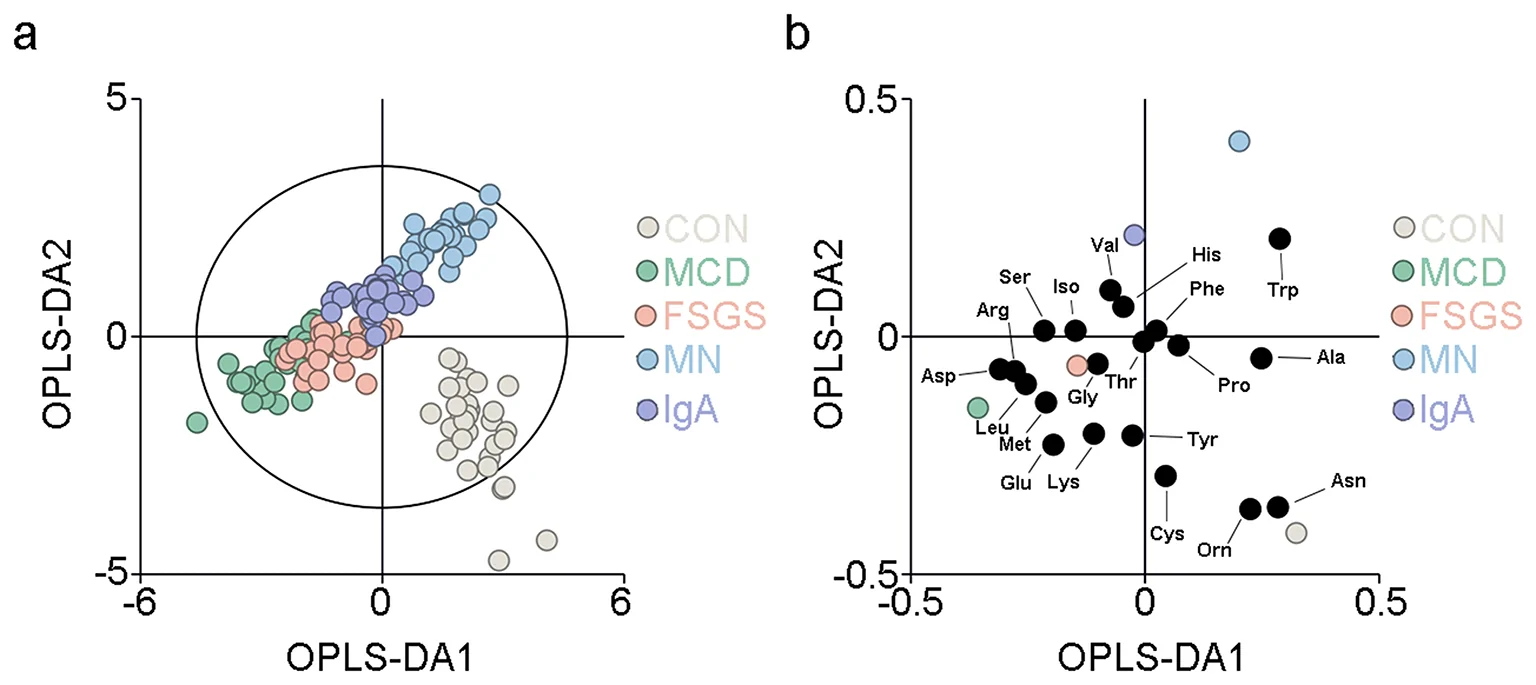
Metabolomic profiles of the 20 amino acids (AAs) in plasma in the MCD group, FSGS, group, MN group, IgAN group and CON group. (**a**) Orthogonal partial-least-squares discriminant analysis (OPLS-DA) score plot shows the visual separation of the MCD group, FSGS, group, MN group, IgAN group and CON group. The ellipse indicates the Hotelling T2 (0.95) range for the model. (**b**) Loading plot analysis showing the relative contributions of the 20 AAs to the differences between the MCD, FSGS, MN, IgAN and CON groups. MCD, minimal change disease; FSGS, focal segmental glomerular sclerosis; MN membranous nephropathy; IgAN IgA nephropathy; CON, healthy controls

Blood samples were collected from the participants with MCD, FSGS, MN, or IgAN between March 2022 and October 2023. The demographic characteristics of the study sample are summarized in Supplementary Table S4. The AA assay protocol was described in our previous publication (Zhou et al. 2021). The plasma concentrations of AAs were quantified using the established UPLC-MS/MS method, which revealed significant differences in the AA profiles of the participants with PGD. In particular, the plasma AA profiles differed between participants with MCD, FSGS, MN, and IgAN (Fig. 1). Next, an OPLS-DA, a supervised multivariate statistical method, was used to visually demonstrate the differences among the control (CON), MCD, FSGS, MN, and IgAN groups (R2X = 0.608, R2Y = 0.752, Q2 = 0.703; Fig. 1). This result was validated using the random permutation test (Supplementary Fig. S1).

**Fig. 2:**
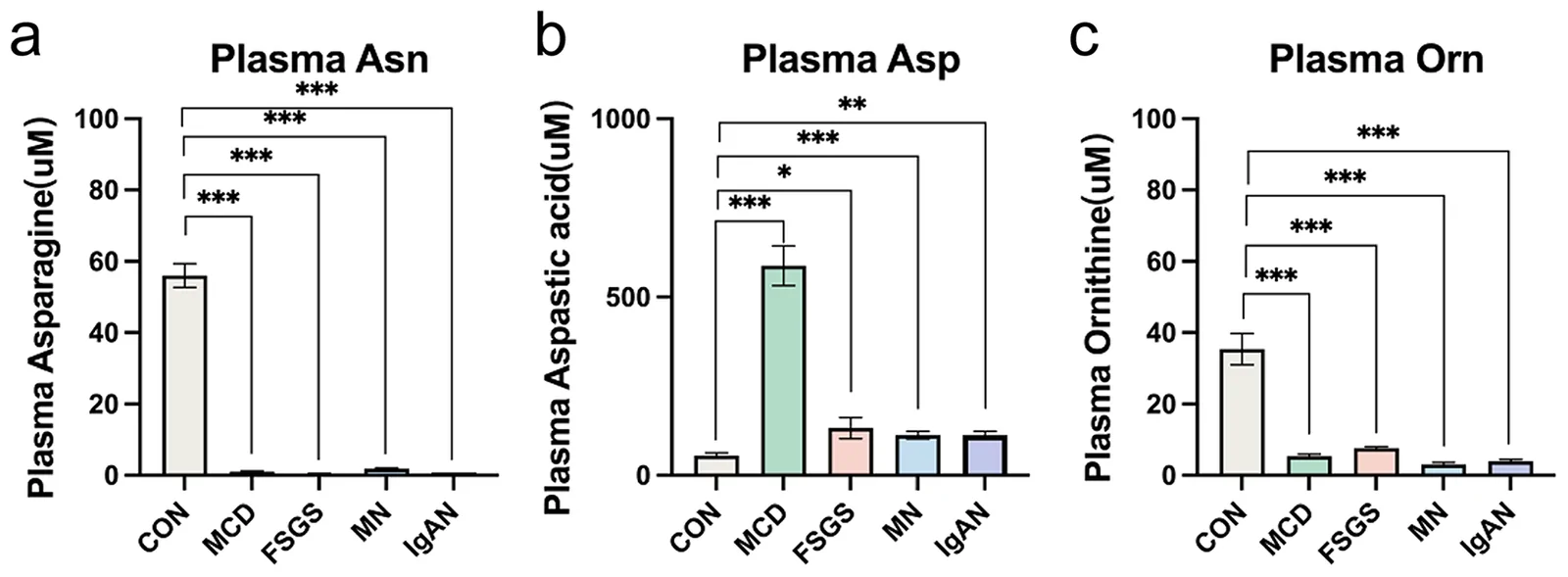
Plasma levels of Asparagine, Aspartic acid and Ornithine. Plasma levels of (**a**) asparagine, (**b**) aspartic acid and (**c**) ornithine in the CON, MCD, FSGS, MN, and IgAN groups, respectively. *P < 0.05, **P < 0.01, ***P < 0.001

The plasma concentrations of 13 of the 20 AAs in the significantly differed in the MCD or FSGS groups vs. the CON group (*P* < 0.05). In addition, 11 of the 20 AAs in the MN group and 12 of the 20 AAs in the IgAN group significantly differed in their concentrations from those in the CON group (*P* < 0.05). More specifically, the plasma concentrations of asparagine (Asn), aspartic acid (Asp), and ornithine (Orn) showed the same trends in patients with the various pathologic types of PGD. Significantly lower plasma Asn and Orn concentrations (*P* < 0.001) and a significantly higher plasma concentration of Asp (*P* < 0.001) were present in patients with PGD vs. controls (Fig. 2).

### Plasma amino acid signatures for participants with MCD, FSGS, MN, and IgAN

**Fig. 3:**
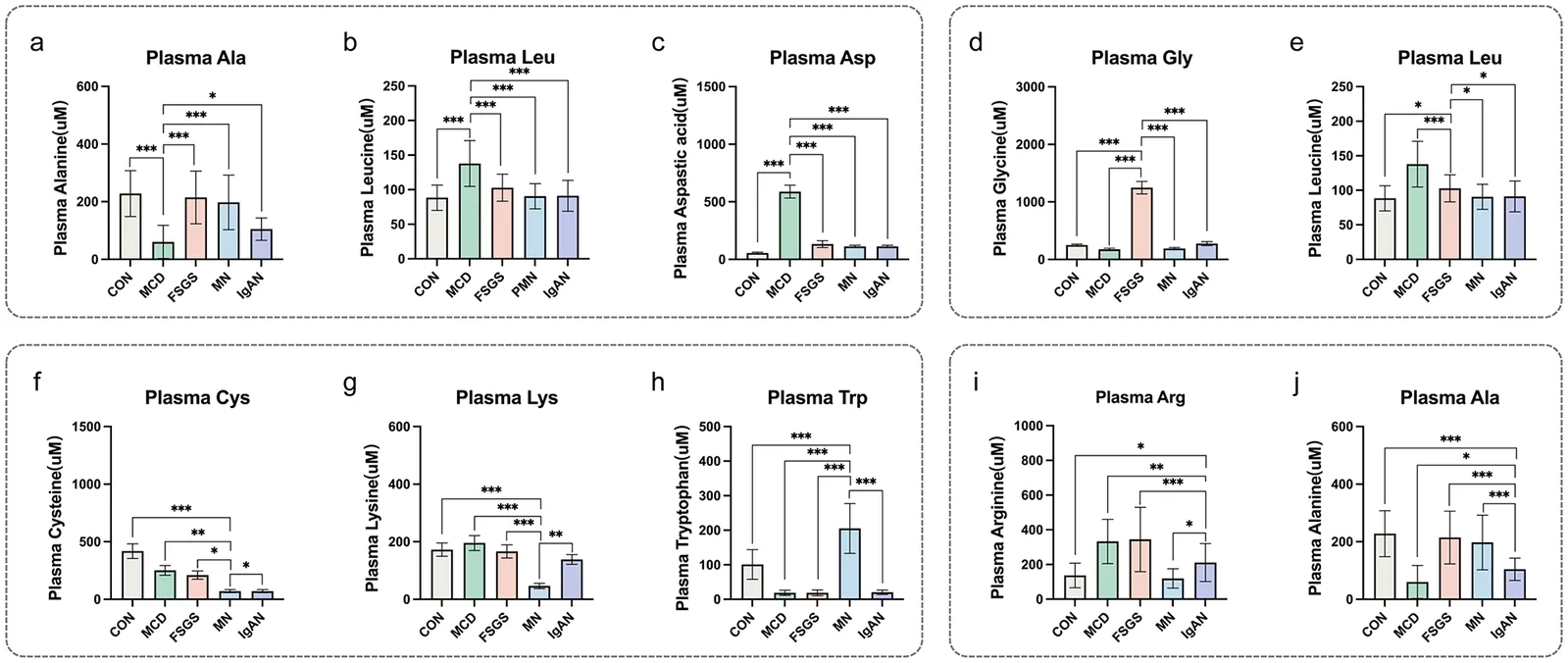
Plasma levels of amino acids distinguishing MCD, FSGS, MN, and IgAN groups. Plasma levels of (**a**) alanine, (**b**) leucine, (**c**) aspartic acid, (**d**) glycine, (**e**) leucine, (**f**) cysteine, (**g**) lysine, (**h**) tryptophan, (**i**)arginine and (**j**) alanine in the CON, MCD, FSGS, MN, and IgAN groups, respectively. *P < 0.05, **P < 0.01, ***P < 0.001

The plasma concentrations of alanine (Ala), leucine (Leu), and Asp in the MCD group significantly differed from those of the other PGD or CON groups (*P* < 0.05) (Supplemental Table 4). Specifically, there was a significantly lower Ala concentration (*P* < 0.05) and higher concentrations of Leu and Asp (*P* < 0.05) in the MCD group (Fig. 3).

The plasma concentrations of glycine (Gly) and Leu in the FSGS group significantly differed from those in the other PGD or CON groups (*P* < 0.05) (Supplemental Table 4). Specifically, there was a significantly higher concentration of Gly (*P* < 0.05) in the FSGS group, and a lower concentration of Leu in the FSGS group than in the MCD group, but a higher concentration than in the MN, IgAN, or CON groups (*P* < 0.05) (Fig. 3).

The plasma concentrations of cysteine (Cys), lysine (Lys), and tryptophan (Trp) in the MN group significantly differed from those in the other PGD or CON groups (*P* < 0.05) (Supplemental Table 4). Specifically, there were significantly lower concentrations of Cys and Lys (*P* < 0.05) and a higher concentration of Trp (*P* < 0.05) in the MN group (Fig. 3).

The concentrations of Ala and arginine (Arg) in the IgAN group significantly differed from those in the other PGD or CON groups (*P* < 0.05) (Supplemental Table 4). The Ala concentration was lower in the IgAN group than in the FSGS, MN, or CON groups, but higher than in the MCD group (*P* < 0.05) (Fig. 3).

### Development and validation of the diagnostic models

**Fig. 4:**
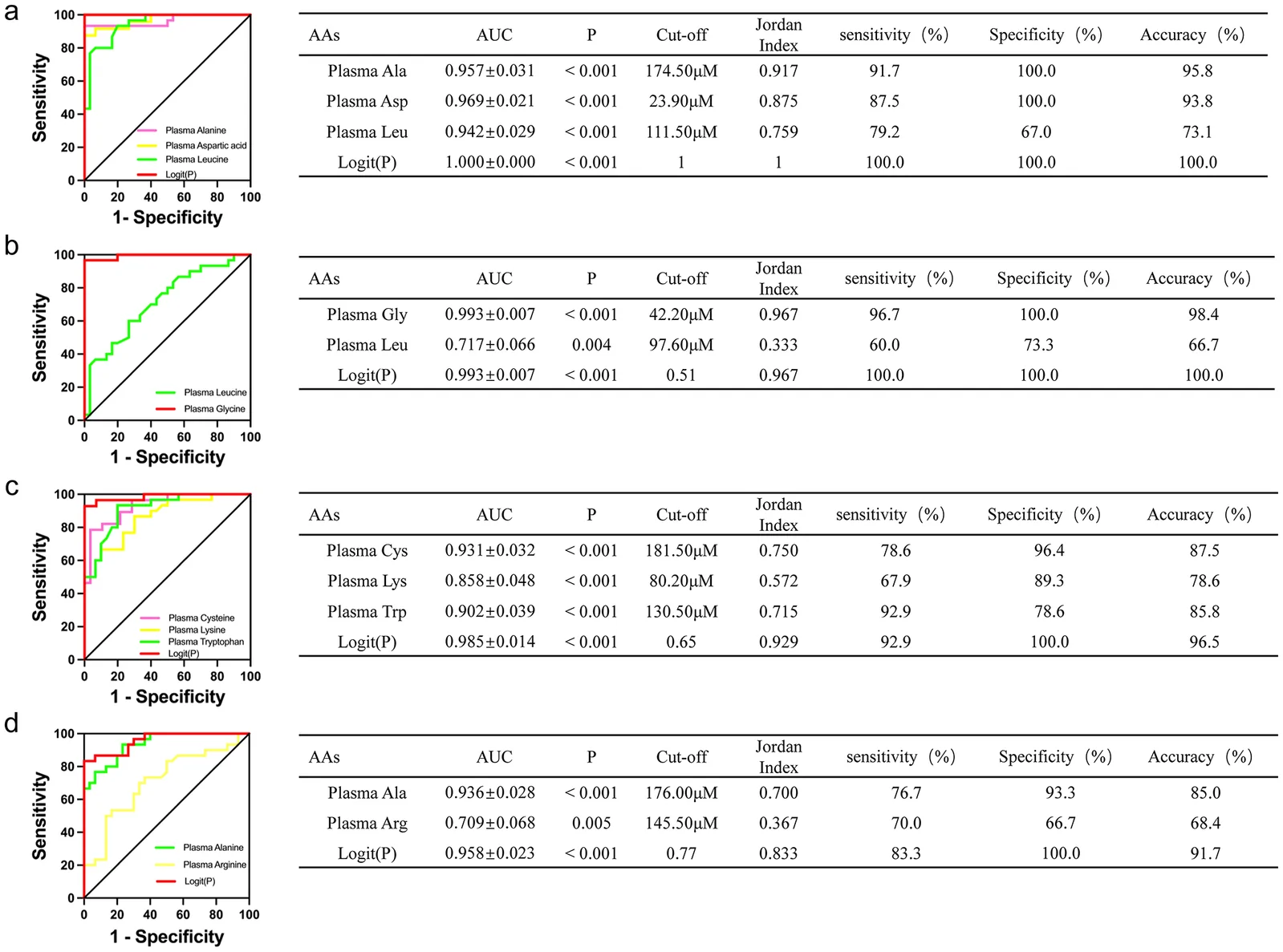
ROC curve and AUC for different model. (**a**) alanine, aspartic acid, leucine and Logit(P); (**b**) glycine, leucine and Logit(P); (**c**) cysteine, lysine, tryptophan and Logit(P); (**d**)alanine, arginine and Logit(P). Accuracy = (A×sensitivity + B×specificity) / (A + B), where A is the number of participants in the corresponding disease group and B is the number of participants in the corresponding control group

To distinguish participants with different pathologic diagnoses of PGD, logistic regression was performed and the diagnostic models were established on the basis of the differing plasma AA concentrations in the participants in the MCD, FSGS, MN, IgAN, and CON groups. For the MCD group, the ROC curve had an AUC of 1 (95% CI, 1.000–1.000), with a Youden index J of 1 (Fig. 4 and Supplemental Table 5), indicating that the predictive performance of the diagnostic model was excellent, with an accuracy of up to 100.0% (Fig. 4).

For the FSGS group, the ROC curve had an AUC of 0.993 (95% CI, 0.979–1.000), with a Youden index J of 0.51 (Fig. 4 and Supplemental Table 6), indicating excellent predictive performance of the diagnostic model, with an accuracy of up to 100.0% (Fig. 4).

For the MN group, the ROC curve had an AUC of 0.985 (95% CI, 0.958–1.000) with a Youden index J of 0.65 (Fig. 4 and Supplemental Table 7), indicating excellent predictive performance of the diagnostic model, with an accuracy of 96.5% (Fig. 4).

For the IgAN group, the ROC curve had an AUC of 0.958 (95% CI, 0.914–1.000), with a Youden index J of 0.77 (Fig. 4 and Supplemental Table 8), indicating excellent predictive performance of the diagnostic model, with an accuracy of 91.7% (Fig. 4).

## Discussion

In the present study, we have shown that the plasma concentrations of Asn and Orn are significantly lower, and that of Asp is significantly higher, in patients with any of the pathologic types of PGD, including MCD, FSGS, MN, and IgAN, vs. healthy individuals. Asp is a glutamine-derived metabolite for proliferating cells that sustains cellular homeostasis and plays important roles in blood vessel formation(Huang et al. 2017) and the regulation of protein and nucleotide synthesis(Krall et al. 2016). Asp has been reported to be an important contributor to cancer cell growth and may underpin novel strategies to improve the efficacy of cancer treatments. To date there have been few studies of the relationship between the plasma Asp concentration and kidney disease. Here, we have shown for the first time that the plasma Asp concentrations of patients with any of the pathologic types of PGD are higher than those of healthy individuals.

The Orn cycle is a critical AA metabolism pathway by which mammals dispose of waste nitrogen(Morris 2002), and it is associated with multiple metabolic processes. For example, in the tricarboxylic acid cycle, oxaloacetic acid is converted to Asp, which provides an important nitrogen source for urea production by the ornithine cycle(Posset et al. 2019). Thus, Asp is an important carrier of nitrogen atoms for the urea cycle, in which Orn is an intermediate. Dysregulation of the Orn cycle affects the regulation of several other metabolic processes, leading to hyperornithinemia and hyperammonemia(Sivashanmugam et al., 2017), and is a feature of many diseases, including infections(Lercher et al. 2019), cancer(Lee et al. 2018), and hypertension(Zheng et al. 2018). Furthermore, a previous study showed that the renal concentrations of Asp are increased by the injection of Orn, which may be the result of interference with the renal urea cycle(Li et al. 2021).

Asp is an essential AA that has been widely studied by researchers into kidney disease. Chen et al. analyzed the AA metabolites in the plasma of rats with CKD, and showed that they had significantly higher circulating concentrations of Asp than controls, and these correlated with their circulating creatinine concentrations. Further investigations showed that Asp may represent a useful biomarker for chronic interstitial kidney disease(Chen et al. 2017). Hirayama et al. also found that high serum Asp concentrations are associated with the diagnosis and severity of nephropathy in patients with diabetes, and therefore proposed that Asp may also represent a useful serum biomarker of diabetic nephropathy(Hirayama et al. 2012). Interestingly, another study demonstrated that the circulating Asp concentrations of diabetic mice are low, and that Asp supplementation might be useful to prevent the progression of DKD, through the amelioration of endothelial function(Elitok et al. 2006). In the present study, we found that the plasma Asp concentration is high in the patients with any of the pathologic types of PGD, which is consistent with the findings of previous studies. This high Asp concentration may increase the efficiency of the Orn cycle, resulting in greater urea excretion, and leading to a decrease in the Orn concentration(Das et al. 2020).

PGD is usually diagnosed on the basis of the histologic features of an invasive puncture biopsy sample, the collection of which is associated with the risks of hematuria or perirenal hematoma(Stiles et al. 2000). The lack of suitable early detection methods is a major obstacle to the prevention and treatment of CKD. Therefore, in the present study, we used a targeted metabolomic approach to evaluate the plasma AA concentrations of patients and controls, and identified a different AA profile in patients with PGD.

To assess the diagnostic utility of the assessment of AAs for the diagnosis of the different pathologic types of PGD, we conducted a logistic regression analysis of the MCD, FSGS, MN, and IgAN groups to generate four optimal diagnostic models. Simple and accurate MCD and FSGS diagnostic models were then established. The AUCs for the MCD and FSGS diagnostic models were 1.000, and the ROCs were associated with 100.0% specificity, sensitivity, and accuracy. The excellent performance of the MCD diagnostic model was principally the result of the high Asp concentration and the low Ala concentration in the MCD group vs. the FSGS, MN, and IgAN groups. Previous studies of the plasma concentration of Ala have principally been focused on its relationship with the aberrant metabolism and complications associated with diabetes, and these showed that there may be protective effects of Ala against the complications of diabetes(Adachi et al. 2018; Hou et al. 2021; Welsh et al. 2018). However, only a few studies have been performed to investigate the metabolism of Ala in kidney disease. In the present study, we have demonstrated for the first time that the plasma Asp concentrations are high and the Ala concentrations are low in patients with MCD confirmed by renal pathology, and therefore these AAs may represent useful biomarkers for the diagnosis of MCD. Furthermore, because the plasma Gly concentration in patients with FSGS is higher than the concentrations in patients with MCD, MN, or IgAN, the diagnostic model for this condition was also found to be very accurate.

FSGS is the pathologic type of PDG that is associated with the highest probability of progression to ESRD(Sim et al. 2018). The main pathologic lesions are in the glomeruli (focal) and glomerular bundles (segmental), along with sclerosis and tubulointerstitial fibrosis(Wilkening et al. 2020). Renal fibrosis is the result of an imbalance between collagen synthesis and degradation(Hijmans et al. 2017; McKleroy et al. 2013). Collagen is the most abundant structural protein in mammals, and more than half of this protein is composed of Gly, hydroxyproline, and Pro, with 30% being composed of Gly alone(Li and Wu 2018; Wang et al. 2006). McMahon et al. found that CKD is associated with a low urinary Gly concentration, but no correlation was identified between the plasma Gly concentration and the progression of CKD(McMahon et al. 2017). Gly has also been shown to have beneficial anti-inflammatory, immune regulatory, and cytoprotective effects(Zhong et al. 2003). In the present study, we have shown for the first time that the plasma Gly concentration is high in patients with FSGS, and therefore this may represent a useful biomarker for the diagnosis of FSGS.

Some limitations to the present study should be mentioned. First, because the present study was cross-sectional in nature, the potential causal relationship between PGD and AAs should be further explored. Second, the sample size of the study was relatively small. Third, we performed targeted metabolomic profiling of 20 AAs, whereas a combination of untargeted and targeted metabolomic studies may have permitted the generation of clearer AA profiles for the various pathologic types of PGD. Therefore, further, larger investigations, including prospective longitudinal studies and in vivo or in vitro intervention studies, are needed to further explore the relationship between AA metabolism and the progression of PGD.

In conclusion, using a UPLC-MS/MS-based metabolomic approach, we have demonstrated, for the first time, that differences in the plasma concentrations of AAs could be used to identify patients with the various pathologic types of PGD. We characterized the plasma profiles of 20 AAs from patients with PGD, and found that high plasma concentrations of Asp and low concentrations of Asn and Orn characterize patients with PGD, which may provoke a new avenue of research into the pathogenesis of PGD. We then established four distinct diagnostic models based on these AAs, and the accuracy of the models for MCD and FSGS were as high as 100.0%. In summary, our findings provide a theoretical basis for the use of AAs as non-invasive, real-time, rapid, and simple biomarkers for the diagnosis and prediction of the prognosis of various pathologic types of PGD.

## Electronic supplementary material

Below is the link to the electronic supplementary material.

Supplementary Material 1

## Data Availability

No datasets were generated or analysed during the current study.
