# Supplementary material for: Differentiated metabolomic profiling reveals plasma amino acid signatures for primary glomerular disease
Source: Amino Acids. 2024 Jul 18;56(1):46. doi: 10.1007/s00726-024-03407-4 (PMC11255010; doi:10.1007/s00726-024-03407-4)
Supplement: Supplementary file 1 — Supplementary Material 1 [file 726_2024_3407_MOESM1_ESM.docx]

Supplementary Material

**Differentiated metabolomic profiling reveals plasma amino acid signatures for primary glomerular disease**

Jiao Wang, Chunyu Zhou, Liqian Lu, Shoujun Wang, Qing Zhang*, Zhangsuo Liu*

*** Correspondence:** Dr. Qing Zhang: aquamarine61@163.com

Dr.Zhangsuo Liu: zhangsuoliu@zzu.edu.cn. Tel.: +86-0371-66271018

# Supplementary Data

*Reagents*

The standard compounds of 20 amino acids (AAs) were purchased from MedChem Express (Monmouth Junction, NJ, USA); the corresponding isotope-labeled AA mix was purchased from Sigma-Aldrich (St. Louis, MO, USA; #909653). Details of these standards are listed in Supplemental Tables 1 and 2. Acetonitrile (ACN, #A998-4), methanol (MeOH, #A452-4), and water (#34877) were purchased from Thermo Fisher Scientific (Waltham, MA, USA); 2-mL capped vials (#5183-4331) were from Agilent Technologies (Santa Clara, CA, USA); 1-methylhistidine (1-MH) (#67520; Sigma-Aldrich), 3-methylhistidine (3-MH) (#M9005; Sigma-Aldrich), anserine (#B24424; Yuanye Bio-Technology Co., Shanghai, China), carnosine (#HY-W013494; MedChem Express), ergothioneine (#HY-N1914; MedChem Express), homocarnosine (S182442; Sage Chemical, Hangzhou, China), trans-urocanate (SU8120; Solarbio Science & Technology, Beijing, China), and 3-methyl-2-oxobutyrate (#169975; Aladdin, Shanghai, China) were purchased from local distributors.

*Preparation of plasma, urine and saliva samples for metabolic profiling*

For sample preparation, we transferred 50 μL of thawed plasma and 50 μL of ACN to a 1.5-mL tube containing 150 μL of internal standard (IS) (100 nM), and standard curves were generated by mixing 50 µL of plasma (mixed from 30 healthy individuals), 50 µL of working solution, and 150 µL of IS. We mixed all samples by vortexing for 10 min, followed by centrifugation at 4℃ and 15,000 rpm. The supernatants were transferred into 250-µL inserts fixed in 2-mL vials for further metabolic analyses.

*UPLC-MS/MS parameters*

[M+H]^+^ precursor ions were used for AAs and ISs. The optimized MS parameters of compounds were the following: capillary voltage, 5500 V; source temperature, 550°C; curtain gas (N_2_), 30 psi; collision gas, 10 psi; pressure for nebulization gas, 40 psi; evaporization gas; 40 psi; entrance potential, 50 V; and, collision cell exit, 10 V. The Tables S1 and S2 list the detailed declustering potential and collision energy as well as the precursor and dominant daughter ions of AAs and ISs.

Chromatographic separation was performed at 40℃ using an UPLC BEH amide column (2.1×100 mm, 3-μm particle size; Waters, Milford, MA, USA) equipped with a 1.7-μm VanGuard precolumn; the isocratic gradient elution program (Table S3) was run with mobile phase B (ACN) and mobile phase A (water containing 0.1% formic acid and 0.05% trifluoroacetic acid).

# Supplementary Tables and Figures

## Supplementary Tables

**Supplemental Table 1.** MS parameters, measurement range, and quantification of 20 amino acids

| **Amino acid** | **MS number** | **Abbreviation** | **ID** | **CAS number** | **Catalog ID^#^** | **MRM transition** | **DP (V)** | **CE (V)** | **Range (nM)** | **Linearity (R^2^)** |
| --- | --- | --- | --- | --- | --- | --- | --- | --- | --- | --- |
| Glycine | a | Gly | HMDB0000123 | 56-40-6 | HY-N0390 | 76→30 | 20 | 18 | 10-1000 | 0.9985 |
| L-Alanine | b | Ala | HMDB0000161 | 56-41-7 | HY-N0229 | 90→44 | 23 | 20 | 10-1000 | 0.9999 |
| L-Arginine | m | Arg | HMDB0000517 | 74-79-3 | HY-N0455 | 175→70 | 38 | 23 | 50-1000 | 0.9906 |
| L-Asparagine | c | Asn | HMDB0000168 | 70-47-3 | HY-N0667 | 133→116 | 35 | 13 | 10-1000 | 0.9951 |
| L-Aspartic acid | d | Asp | HMDB0000191 | 56-84-8 | HY-N0666 | 134→74 | 40 | 17 | 10-1000 | 0.9973 |
| L-Cysteine | e | Cys | HMDB0000574 | 52-90-4 | HY-Y0337 | 122→76 | 30 | 17 | 10-1000 | 0.9981 |
| L-Glutamic acid | f | Glu | HMDB0000148 | 56-86-0 | HY-14608 | 148→102 | 85.7 | 15 | 50-1000 | 0.9975 |
| L-Histidine | n | His | HMDB0000177 | 71-00-1 | HY-N0832 | 156→110 | 30 | 20 | 50-1000 | 0.9941 |
| L-Isoleucine | o | Ile | HMDB0000172 | 73-32-5 | HY-N0771 | 132→86 | 30 | 14 | 10-1000 | 0.9901 |
| L-Leucine | g | Leu | HMDB0000687 | 61-90-5 | HY-N0486 | 132→30 | 40 | 20 | 20-1000 | 0.9912 |
| L-Lysine | p | Lys | HMDB0000182 | 56-87-1 | HY-N0469 | 147→84 | 30 | 23 | 50-1000 | 0.9965 |
| L-Methionine | q | Met | HMDB0000696 | 63-68-3 | HY-N0326 | 150→133 | 24 | 12 | 10-1000 | 0.9978 |
| L-Ornithine | h | Orn | HMDB0000214 | 70-26-8 | HY-B1352 | 133.1→70.3 | 41 | 20 | 10-1000 | 0.9959 |
| L-Phenylalanine | r | Phe | HMDB0000159 | 63-91-2 | HY-N0215 | 166→120 | 40 | 20 | 10-1000 | 0.9977 |
| L-Proline | s | Pro | HMDB0000162 | 147-85-3 | HY-Y0252 | 116→70 | 60 | 27 | 10-1000 | 0.9933 |
| L-Serine | i | Ser | HMDB0000187 | 56-45-1 | HY-N0650 | 106→60 | 27 | 15 | 10-1000 | 0.9906 |
| L-Threonine | j | Thr | HMDB0000167 | 72-19-5 | HY-N0658 | 120→74 | 30 | 14 | 10-1000 | 0.9981 |
| L-Tryptophan | k | Trp | HMDB0000929 | 73-22-3 | HY-N0623 | 205→146 | 23 | 23 | 20-1000 | 0.9935 |
| L-Tyrosine | l | Tyr | HMDB0000158 | 60-18-4 | HY-N0473 | 182→136 | 36 | 17 | 10-1000 | 0.9964 |
| L-Valine | t | Val | HMDB0000883 | 72-18-4 | HY-N0717 | 118→72 | 40 | 13 | 10-1000 | 0.9964 |

^#^Catalog ID corresponding to amino acids purchased from MedChem Express (Monmouth Junction, NJ, USA).

AA, amino acid; CE, collision energy; DP, declustering potential; MRM, multiple reaction monitoring; MS, mass spectrometry; nM, nanomole.

**Supplemental Table 2.** MS parameters of 20 isotope-labeled amino acids used as internal standards

| **Chemical** | **MRM transition** | **DP (V)** | **CE (V)** |
| --- | --- | --- | --- |
| Glycine-13C2,15N | 79→32.1 | 40 | 20 |
| L-Alanine-13C3,15N | 94.1→47.1 | 40 | 30 |
| L-Arginine-13C6,15N4 | 185.1→75.2 | 40 | 30 |
| L-Asparagine-15N2 | 135.1→75 | 80 | 30 |
| L-Aspartic acid-13C4,15N | 139.1→77 | 30 | 30 |
| L-Cystine-3,3,3′,3′-d4 | 245.2→122.2 | 30 | 25 |
| L-Glutamic acid-13C5,15N | 154→89.1 | 30 | 25 |
| L-Histidine-15N3 | 159→113 | 30 | 20 |
| L-Isoleucine-13C6,15N | 139→92.1 | 60 | 30 |
| L-Leucine-13C6,15N | 138.1→91 | 30 | 20 |
| L-Lysine-13C6,15N2 | 155.1→90.1 | 40 | 25 |
| L-Methionine-15N | 156→109.1 | 65 | 20 |
| L-Glutamine-15N2 | 149→131 | 40 | 15 |
| L-Phenylalanine-13C9,15N | 176.1→129 | 40 | 20 |
| L-Proline-13C5,15N | 122.1→75.1 | 30 | 20 |
| L-Serine-13C3,15N | 110.1→63 | 40 | 20 |
| L-Threonine-13C4,15N | 125.1→78.1 | 20 | 20 |
| L-Tryptophan-15N2 | 207.1→189.1 | 50 | 15 |
| L-Tyroxine-13C9,15N | 192.1→130.1 | 50 | 25 |
| L-Valine-13C5,15N | 124.1→77.1 | 25 | 20 |

CE, collision energy; DP, declustering potential; MRM, multiple reaction monitoring; MS, mass spectrometry.

**Supplemental Table 3.** Gradient program for liquid chromatography

| **Total time (min)** | **Flow rate (μl/min)** | **A^a^ (v/v, %)** | **B^b^ (v/v, %)** |
| --- | --- | --- | --- |
| 0.00 | 300 | 99.0 | 1 |
| 1.50 | 300 | 98.0 | 2 |
| 2.50 | 300 | 80.0 | 20.0 |
| 3.00 | 300 | 5.0 | 95.0 |
| 4.50 | 300 | 5.0 | 95.0 |
| 5.00 | 300 | 99.0 | 1.0 |
| 8.00 | 300 | 99.0 | 1.0 |

^a^Solvent A, water containing 0.1% formic acid (v/v) and 0.05% trifluoroacetic acid; ^b^Solvent B, acetonitrile.

**Supplemental Table 4.** Plasma levels of the 20 amino acids in study participants.

| **AA**  **(μM)** | **CON**  **(n=30)** | | **MCD**  **(n=30)** | | **FSGS**  **(n=30)** | **MN**  **(n=30)** | **IgAN**  **(n=30)** | | | | | ***P1*** | | | | ***P2*** | ***P3*** | | ***P4*** | ***F*** | |  |
| --- | --- | --- | --- | --- | --- | --- | --- | --- | --- | --- | --- | --- | --- | --- | --- | --- | --- | --- | --- | --- | --- | --- |
| Gly | 251.4±82.3  (120.7-404.1) | | 177.1±101.6  (64.9-537.3) | | 1248.2±598.4  (330.0-2480.0) | 190.7±98.1  (54.4-438.0) | 275.7±185.8  (64.5-922.8) | | | | | 0.003 | | | | <0.001 | 0.015 | | 0.515 | <0.001 | |  |
| Ala | 228.1±79.9  (113.0-388.0) | | 60.4±57.1  (8.1-217.8) | | 214.8±91.6  (94.1-390.0) | 197.5±94.6  (57.2-485.0) | 104.7±38.4  (41.9-192.0) | | | | | <0.001 | | | | 0.554 | 0.182 | | <0.001 | <0.001 | |  |
| Arg | 136.9±70.5  (51.2-297.0) | | 332.9±127.7  (147.0-615.0) | | 393.3±261.5  (84.1-1230.0) | 119.4±55.8  (38.1-296.0) | 211.1±109.4  (64.8-510.0) | | | | | <0.001 | | | | <0.001 | 0.289 | | 0.003 | <0.001 | |  |
| Asn | 56.0±18.3  (14.4-98.6) | | 1.1±0.8  (0.3-4.2) | | 0.5±0.2  (0.3-1.2) | 1.8±1.1  (0.8-4.9) | 0.8±0.09  (0.7-1.1) | | | | | <0.001 | | | | <0.001 | <0.001 | | <0.001 | <0.001 | |  |
| Asp | 55.4±41.9  (9.5-189.0) | | 587.9±274.3 (54.5-955.0) | | 132.6±161.4  (11.4-654.0) | 112.7±59.6  (20.9-250.6) | 114.3±89.7  (13.6-350.9) | | | | | <0.001 | | | | 0.014 | <0.001 | | 0.002 | <0.001 | |  |
| Cys | 433.9±335.6  (48.8-1060.0) | | 290.4±225.1 (14.4-920.8) | | 211.2±188.4  (47.3-854.0) | 70.9±71.0  (1.2-250.0) | 200.3±233.3  (10.3-123.0) | | | | | 0.073 | | | | 0.003 | <0.001 | | 0.004 | <0.001 | |  |
| Glu | 483.7±429.4  (99.6-2270.0) | 955.8±979.2 (88.0-4080.0) | | 699.1±415.7  (41.5-1620.0) | | 40.2±42.8  (1.3-5156.0) | 132.5±107.6  (39.5-489.0) | | | | | 0.019 | | | | 0.043 | <0.001 | | <0.001 | <0.001 | |  |
| His | 29.0±10.1  (15.0-50.7) | 32.4±15.1 (10.1-70.0) | | 37.8±10.3  (20.5-58.8) | | 34.9±15.5  (13.0-67.9) | | | | 33.6±13.9  (13.4-60.6) | | 0.314 | | | | 0.002 | 0.085 | | 0.156 | 0.127 | | |
| Iso | 84.9±20.6  (47.5-133.0) | 109.9±28.8 (52.0-176.0) | | 103.8±25.5  (63.3-172.0) | | 92.8±22.2  (61.9-153.0) | | | | 100.4±27.2  (53.5-168.0) | <0.001 | | | | | 0.003 | 0.156 | | 0.015 | 0.002 | | |
| Leu | 88.3±18.3  (49.7-140.0) | 137.8±33.1 (93.4-233.0) | | 102.9±19.6  (64.0-144.0) | | 90.5±18.2  (59.6-131.0) | | | | 91.2±22.2  (57.3-145.0) | <0.001 | | | | 0.004 | | 0.646 | 0.595 | | | <0.001 | |
| Lys | 172.7±124.9 (36.4-438.0) | 195.6±140.6 (34.5-490.0) | | 166.5±123.6  (42.7-511.0) | | 46.6.7±51.3  (2.6-252.0) | | | | 138.8±92.8  (20.5-375.0) | 0.508 | | | | 0.848 | | <0.001 | 0.238 | | | <0.001 | |
| Met | 32.9±10.7  (13.5-54.4) | 49.0±14.9 (26.9-83.4) | | 45.3±16.3  (20.5-96.5) | | 29.2±6.0  (15.3-38.1) | | | | 30.0±7.6  (15.0-46.3) | <0.001 | | | | 0.001 | | 0.096 | 0.229 | | | <0.001 | |
| Orn | 35.4±24.0  (8.5-119.0) | 5.4±2.9  (2.0-14.0) | | 7.6±1.9  (4.1-13.2) | | 3.1±2.8  (0.5-12.8) | | | | 3.9±2.7  (0.9-11.1) | <0.001 | | | | <0.001 | | <0.001 | <0.001 | | | <0.001 | |
| Phe | 58.6±10.6  (42.4-86.4) | 57.6±13.9 (34.4-83.6) | | 56.7±11.3  (40.3-78.3) | | 57.4±11.1  (37.0-87.3) | | | | 57.5±13.7  (41.0-81.1) | 0.749 | | | | 0.495 | | 0.665 | 0.722 | | | 0.983 | |
| Pro | 177.4±79.6 (96.4-446.0) | 139.9±66.8 (67.3-340.0) | | 145.7±41.9  (62.2-230.0) | | 140.3±42.8  (43.3-248.0) | | | | 181.4±61.6  (94.6-337.0) | 0.053 | | | | 0.059 | | 0.029 | 0.828 | | | 0.009 | |
| Ser | 51.1±44.8  (6.5-223.0) | 130.1±75.9  (38.1-289.0) | | 106.0±78.1  (23.2-351.0) | | 58.9±59.1  (18.3-347.0) | | | | 126.1±105.5  (24.7-570.0) | <0.001 | | | | 0.001 | | 0.565 | 0.001 | | | <0.001 | |
| Thr | 49.5±12.6  (24.3-72.6) | 47.1±16.2 (24.1-92.4) | | 48.9±16.1  (20.1-87.1) | | 45.9±14.6  (20.1-95.8) | | 45.0±15.5  (24.6-85.0) | | | | 0.522 | | | 0.887 | | 0.316 | 0.221 | | | 0.742 | |
| Trp | 100.9±42.9  (44.2-201.0) | 18.9±7.6  (7.3-35.9) | | \| 18.9±8.3  (8.6-38.7) \| \| --- \| | | 205.3±71.9  (82.8-383.0) | | \| 20.7±6.2  (10.9-32.1) \| \| --- \| | | | | <0.001 | | | <0.001 | | <0.001 | <0.001 | | | <0.001 | |
| Tyr | 38.1±10.7  (20.8-60.6) | 38.1±12.1 (20.1-66.2) | | 33.4±8.7  (20.4-53.2) | | 29.4±6.4  (16.4-46.3) | | 29.9±8.7  (15.8-53.6) | | | | | 0.988 | 0.072 | | | <0.001 | 0.002 | | | <0.001 | |
| Val | 164.3±31.2 (94.0-226.0) | 191.6±43.1 (117.0-294.0) | | 177.5±34.7  (106.0-248.0) | | 189.2±39.9  (133.0-311.0) | | | 189.5±46.3  (111.0-288.0) | | | | 0.007 | 0.129 | | | 0.009 | 0.016 | | | 0.040 | |

Data represent mean ± SD (range). F-values were determined by the one-way ANOVA test; P-values were determined by the Student’s t-test. *P1*: MCD vs CON group; *P2*: FSGS vs CON group; *P3*: MN vs CON group; *P4*: IgAN vs CON group; μM, micromole; CON, healthy controls; MCD, minimal change disease; FSGS, focal segmental glomerular sclerosis; MN membranous nephropathy; IgAN IgA nephropathy; Gly, glycine; Ala, alanine; Arg, arginine; Asn, asparagine; Asp, aspartic acid; Cys, cysteine; Glu, glutamic acid; His, histidine; Iso, isoleucine; Leu, leucine; Lys, lysine; Met, methionine; Orn, ornithine; Phe, phenylalanine; Pro, proline; Ser, serine; Thr, threonine; Try, tryptophan; Tyr, tyrosine; Val, valine.

**Supplemental Table 5.** Logistics regression model parameters of MCD

|  | B | S.E. | Wals | df | sig | Exp（B） |
| --- | --- | --- | --- | --- | --- | --- |
| Plasma Ala | -0.000 | 0.043 | 0.000 | 1 | 0.997 | 1.001 |
| Plasma Asp | -0.001 | 0.240 | 0.000 | 1 | 0.994 | 1.000 |
| Plasma Leu | -0.001 | 0.177 | 0.000 | 1 | 0.995 | 1.001 |
| Constant | -39.906 | 15346.907 | 0.000 | 1 | 0.998 | 0.000 |

**Supplemental Table 6.** Logistics regression model parameters of FSGS

|  | B | S.E. | Wals | df | sig | Exp（B） |
| --- | --- | --- | --- | --- | --- | --- |
| Plasma Gly | 0.000 | 0.000 | 0.011 | 1 | 0.916 | 1.000 |
| Plasma Leu | 0.000 | 0.000 | 4.299 | 1 | 0.038 | 1.000 |
| Constant | -12.452 | 6.875 | 3.280 | 1 | 0.070 | 0.000 |

**Supplemental Table 7.** Logistics regression model parameters of MN

|  | B | S.E. | Wals | df | sig | Exp（B） |
| --- | --- | --- | --- | --- | --- | --- |
| Plasma Cys | 0.000 | 0.000 | 2.543 | 1 | 0.111 | 1.000 |
| Plasma Lys | 0.000 | 0.000 | 5.299 | 1 | 0.021 | 1.000 |
| Plasma Trp | 0.000 | 0.000 | 6.439 | 1 | 0.011 | 1.000 |
| Constant | -0.277 | 1.953 | 0.020 | 1 | 0.887 | 0.758 |

**Supplemental Table 8.** Logistics regression model parameters of IgAN

|  | B | S.E. | Wals | df | sig | Exp（B） |
| --- | --- | --- | --- | --- | --- | --- |
| Plasma Ala | 0.000 | 0.000 | 11.262 | 1 | 0.001 | 1.000 |
| Plasma Arg | 0.000 | 0.000 | 4.677 | 1 | 0.031 | 1.000 |
| Constant | 5.269 | 2.029 | 6.742 | 1 | 0.009 | 194.144 |

## Supplementary Figures


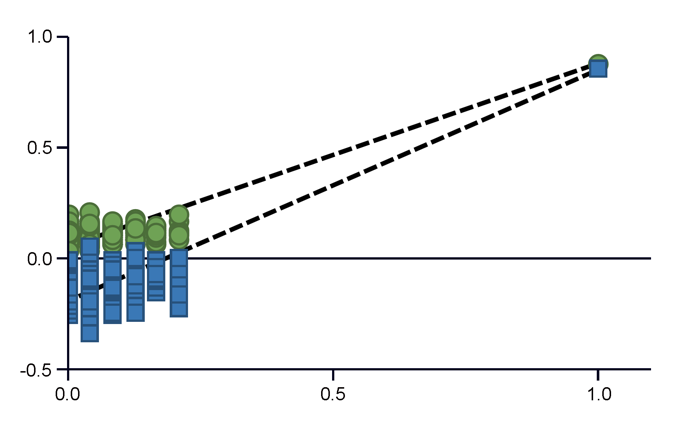


**Supplementary Figure 1. Permutation plots of orthogonal partial least-squares discriminant (OPLS-DA) models.** The permutation indicates the correlation coefficient between the original R^2^ (green dots), Q^2^ (blue dots), and cumulative R^2^, Y^2^; the dashed lines represent the corresponding regression lines. The number of random permutation tests for each plot was 200.
